# Exosomes-based liquid biopsy: An effective approach for drugs and therapeutics resistance screening in cancer

**DOI:** 10.1016/j.jlb.2024.100144

**Published:** 2024-02-05

**Authors:** Sidhanti Nyahatkar, Ketki Kalele

**Affiliations:** a VYWS Dental College & Hospital, WQMV+7X6, Tapovan-Wadali Road, Camp Rd, SRPF Colony, Amravati, Maharashtra, 444602, India; b Neuron Institute of Applied Research, Rajapeth-Irwin Square Flyover, Amrawati Tahsil, Amravati, Maharashtra, 444601, India

**Keywords:** Exosomes, Drug resistance, Therapeutic resistance, microRNA

## Abstract

Cancer drugs and therapeutics resistance are a complicated domain of cancer treatment. Extracellular vesicles (EVs) based cancer investigation reveals several complicated concepts about cancer. EVs are associated with cellular communication. Its bioactive molecular transportation reprograms cellular activity. Exosomes an exciting EVs subpopulation work as masterminds in cancer development and progression. Studies over the past decade have elucidated that exosomes play a vital role in intercellular communication within the tumor microenvironment (TME), influencing multiple events such as hypoxia-induced epithelial-mesenchymal transition (EMT), cancer stemness, angiogenesis, and metastasis. Tumor-derived exosomes (TEXs) associated with drugs and therapeutics resistance development. This article focuses on understanding exosome biology and its implications in cancer drug and therapeutics resistance, exosome-based liquid biopsy application in drug and therapeutic resistance detection, limitations, and future orientation in this domain.

## 1 Introduction

Swift identification and continuous tracking of treatment efficacy could streamline the effective care of cancer patients. To achieve this goal, extensive work has gone into crafting less intrusive blood-based tests, known as liquid biopsies. These assays encompass circulating tumor cells (CTCs), circulating disseminated cells (CDCs), and circulating cell-free nucleic acids (RNAs and DNAs) [1]. A decade ago, the discovery of mRNAs and miRNAs within exosomes from glioblastoma (GBM) [2] and ovarian cancer patients marked a pivotal moment, advocating for exosomes and their contents as cancer biomarkers. Subsequently, a growing body of research has centered on this rapidly advancing field of study. Exosomes function biologically through their bioactive cargo, encompassing lipids, metabolites, proteins, and nucleic acids, which they transport to target cells. Increasing evidence underscores the pivotal role of tumor-derived exosomes (TEXs) in cancer dynamics [[3], [4], [5], [6]]. Both exosomes and their payloads hold potential as cancer prognostic markers, therapeutic focal points, and possibly even as carriers for anticancer medications [7]. Exosomes are initially regarded as “garbage bags” responsible for eliminating waste proteins and metabolites, whereas recently exosomes are been revealed through recent studies to possess biologically active cargo [8], facilitating intercellular communication [9]. Chemotherapeutic resistance poses a significant hurdle in cancer treatment, arising either naturally in tumor cells or evolving during therapy [10]. Acquired resistance emerges from genetic variations impacting drug metabolism and membrane transporters like ABC proteins, expelling drugs from cells and lowering their intracellular levels. Mutations in drug targets also diminish drug efficacy and toxicity. Studies suggest chemotherapy elevates mutation rates in cancer cells, activating survival pathways. Additionally, tumor cells' heightened DNA repair capacity counters drug-induced damage, fostering drug-resistant cell lines. Understanding exosomes involvement in these pathways is crucial in deciphering their role in drug resistance development [11,12]. Exosomes-mediated drug resistance spans various therapies like chemotherapy, radiotherapy, targeted therapy, immunotherapy, and anti-angiogenesis therapy. Diverse mechanisms contribute to this resistance: Exosomes contain drug efflux pumps that sequester anticancer drugs, reducing their concentration within cancer cells [11,12]. Following chemotherapy, these pumps get enriched within exosomes and are shuttled between cells, facilitating drug resistance transmission. Exosomes can also serve as bait, capturing monoclonal antibodies targeting cancer-associated ligands or receptors. Exosomes from drug-resistant cancer cells transfer genetic material and proteins to sensitive cells, fueling drug resistance by epithelial-mesenchymal transition (EMT), altered metabolism, and cancer stemness. Cancer cells actively interact with noncancerous cells in the tumor environment through exosomes, fostering therapy resistance and cancer progression. Investigating exosome-mediated drug resistance remains an evolving research focus. These exosomes cargos from cancer patients biofluids act as therapy response biomarkers. Targeting exosomes has been explored to combat drug resistance by impeding exosomes related signaling pathways. Exosomes unique properties make them promising carriers to tackle drug resistance. In cancer, isolated exosomes molecular analysis was done via several methods which are summarized in Table 1.

**Table 1:** Exosome molecular analysis approaches.

Exosome molecule	Approach	References
DNA	Droplet PCR, Nano-sensor	[46,49]
RNA	qPCR, Nano-sensor	[46,49]
Proteins	Western blot, Mass spectrometry, Flowcytometry, ELISA	[46,47,49]
Lipids	Lipidomic profiling	[46,47,49]
Metabolites	Metabolomic profiling	[46,47,49]

## 2 Role of exosomes in cancer drug resistance

**Fig. 1:**
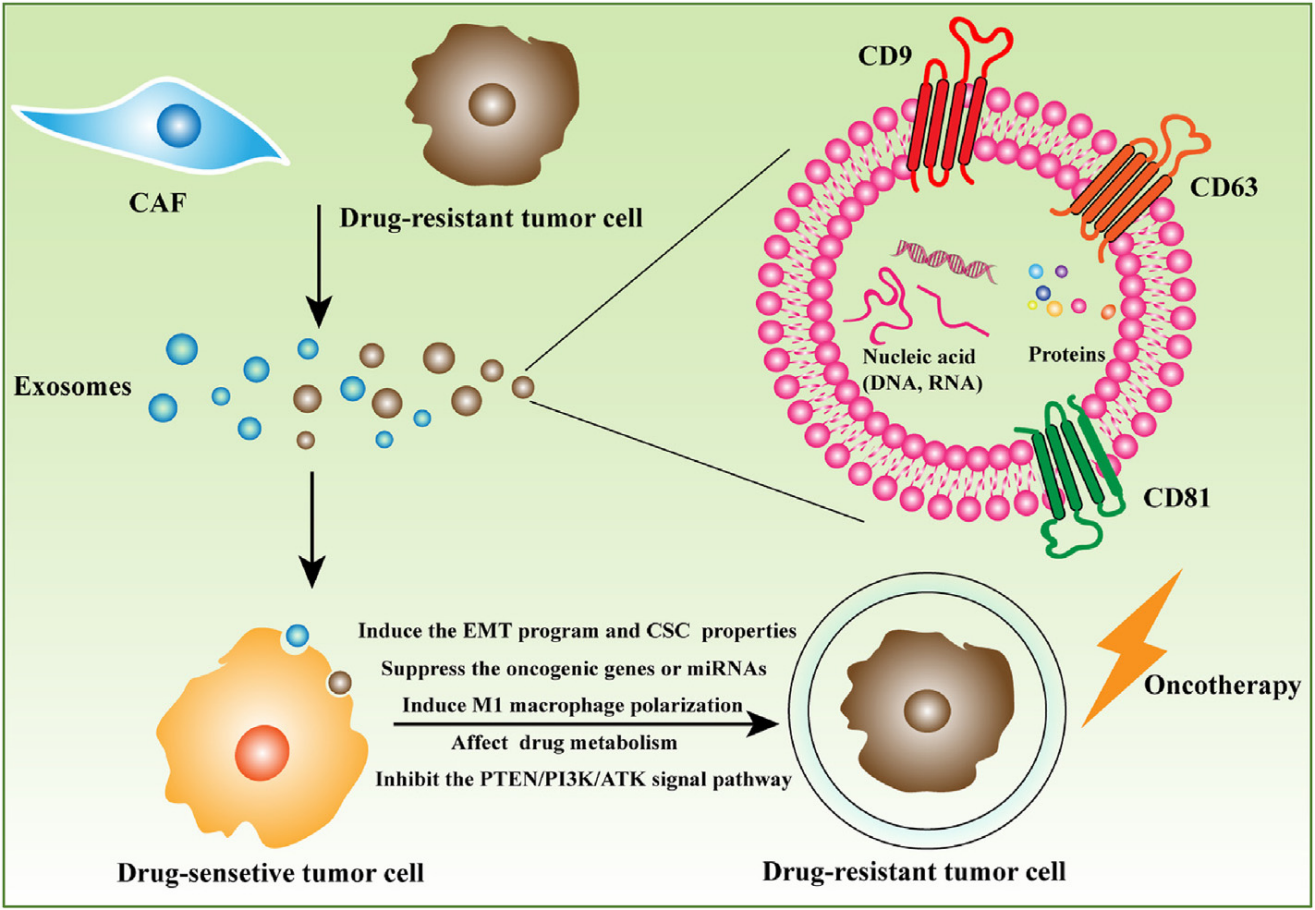
Exosome in cancer drug resistance (Reproduced with permission under Creative Commons CC BY 4.0 license from Ref. [48] Copyright @2020 The Authors).

Resistance to drugs presents a formidable obstacle in cancer treatment, emerging either as an inherent trait or developing after the initial favorable response to therapy. Growing evidence underscores the pivotal role of exosomes in this phenomenon. Exosomes transport specific cargo, such as drug resistance-associated proteins, nucleic acids, and metabolites, either transferring them to cancer cells or actively removing drugs from cells. Cancer-associated fibroblasts (CAFs) derived exosomes linked with cancer drug resistance development (Fig. 1). This process contributes to the emergence of drug resistance. From current research, exosomes emerge as promising nano-carriers capable of reversing tumor drug resistance. For example, Wang *et al.* demonstrated the sensitization of cisplatin-resistant gastric cancer cells by directly delivering anti-miRNA-214 via exosomes to recipient cells [13]. Additionally, interventions like Rapamycin and U18666A sensitize B lymphoma cells to rituximab by inhibiting exosome release, affecting MVB synthesis and cholesterol integration into cell membranes. Moreover, β-elemene manipulates targeted genes in breast cancer cell lines, altering resistance-related miRNA expression in exosomes, consequently reducing resistance transmission and boosting chemotherapy sensitivity [14]. Recent studies reveal that targeting exosomes can prevent and reverse cancer cell chemoresistance. For example, Cao *et al.* demonstrated that GW4869, a neutral sphingomyelinase (NSM) inhibitor, sensitized cisplatin-resistant ovarian cancer cells, highlighting its therapeutic potential in challenging cancer cases [15]. Ketotifen, cannabinol (CBD), and psoralen have been shown to enhance tumor cell sensitivity to chemotherapy by reducing exosome secretion. Similarly, rhamnose-emodin diminishes exosome release from doxorubicin-resistant breast cancer cells, downregulating chemoresistance-associated exosomal miRNAs and reversing drug resistance. Human umbilical cord mesenchymal cell-derived exosomes (hUC-MSC-Exo) sensitize myelogenous leukemia K562 cells to imatinib by activating the caspase signaling pathway, presenting a promising therapeutic strategy against chronic myelogenous leukemia (CML) [16]. Li *et al.* discovered that exosome-specific miRNA-770 reversed doxorubicin resistance in triple-negative breast cancer (TNBC) cells by influencing apoptosis pathways and the tumor microenvironment (TME) [17]. Moreover, Akt inhibitors were effective in reversing chemoresistance instigated by exosomes from drug-resistant cells in sensitive cancer cells [18]. Wang B. *et al.* observed a 2.24-fold increase in the IC50 of cisplatin in chemo-sensitive TNBC cells when co-cultured with a chemo-resistant cell line, which was subsequently reduced upon treatment with the compound Yiqi [19]. Exosomal miRNA-486 and miRNA-10b-5p in saliva indicate head and neck squamous cell carcinoma (HNSCC). Elevated salivary nitrosamines, oxidized DNA/proteins, and reduced antioxidants link oxidative damage to OSCC. Salivary miRNA-21, miRNA-184 in OSCC/PMG highlight non-invasive HNSCC biomarkers. Liquid biopsies, including ctDNA, ctRNA, CTCs, long non-coding RNAs, and exosomal miRNAs, hold clinical potential for HNC. MSC-derived exosomes and exosomal miRNA-8485 impact oral potentially malignant disorders (OPMD) and carcinogenesis. Modifying MSC-derived exosome production could offer innovative approaches to hinder malignant transformation [20]. Exosomes can suppress onco-miRNAs and deliver antagonist tumor-suppressive miRNAs, offering potential in cancer therapy. Strategies involving the removal of exosomes from circulation or preventing their fusion/uptake by target cells serve as therapeutic approaches to impede tumorigenesis. Isolating exosomes from a patient's circulatory system, modifying them, and reintroducing them to the same patient holds promise in cancer treatment [21,22]. Their extended half-life, superior to liposomes, enables specific binding to recipient cell receptors, allowing for the creation of exosomes tailored to target specific cell types.

## 3 Role of exosomes in cancer therapeutic resistance

Exosomes present a potential avenue in genetic therapy, delivering therapeutic agents to specific target cells in specific disorders [23,24]. Certain exo-miRNAs hold promise in inhibiting tumor growth via targeted gene knockdown, serving as nano vectors for delivering anticancer drugs with reduced immunogenicity and toxicity compared to other delivery systems [25,26]. Schmittgen *et al.* highlighted miRNA-186 delivery through natural killer cell-derived exosomes or nanoparticles, restoring natural killer-mediated cytotoxicity and reducing tumor size in neuroblastoma [27]. Experimentation targeting exosomes release or uptake, like Ras-related protein Rab-27A depletion reducing miR-494 abundance, led to decreased tumor growth and metastasis in melanoma [28]. Exosomes effectively deliver antisense oligonucleotides, impeding cell proliferation in leukemia and breast cancer [29]. TEXs involved in therapeutic resistance development in lung cancer [30].

Exosomes delivering antitumor miRNA substantially decreased glioma xenograft growth [31]. They also decreased the expression of multidrug transporters in glioblastoma multiforme, sensitizing cells to temozolomide by inhibiting miRNA-9. Exosomes open a new era in drug delivery systems due to their minimal immunogenicity and remarkable biocompatibility. Their diverse biological functions offer promising prospects in diagnosis, prognosis, and potential therapeutic applications, positioning them as a potential next-generation nano platform for medical use [32].

## 4 Exosomes-based liquid biopsy for drug and therapeutic resistance screening

In the pursuit of more effective cancer screening methods, the conventional approaches have often fallen short in detecting cancer at its early stages. Exosome-based cancer screening, using liquid biopsies, has emerged as a promising technique in this current decade. In cancer patients, biological samples like blood, serum, plasma, and urine contain a mixture of exosomes from both normal and tumor cells. To enable a more specific identification of cancer, the technique of exosome screening has been proposed, and the concept of exosome barcoding has been introduced as a potential solution [33,34]. This approach involves a combination of diverse processes, including exosomes chemical barcoding utilizing fluorescence dyes, Raman dyes, and redox probes, nanoparticle coding employing Quantum dots and plasmonic nanoparticles, biological coding utilizing antibody-DNA conjugates, and physical coding based on optical and electrode assays. Raman dye-based exosome barcoding utilizes nanotags and Raman spectroscopy to detect distinct exosomes released from cancer cells [35]. Fluorescence dye-based barcoding of exosomes involves the use of tags such as aptamers and antibodies [36,37]. This method offers several advantages, including addressing challenges associated with expensive imaging equipment like Magnetic Resonance Imaging (MRI), avoiding invasive procedures for cancer screening, and ensuring high sensitivity. Redox probes, which support high electrocatalytic activity, are employed as a chemical-based method for exosome barcoding [38,39]. This technology, based on nano electrochemistry sensors, aids in the early detection of cancer biomarkers through liquid biopsies. The Quantum dots-based assay for capturing exosomes assists in identifying TEXs based on specific biomarkers. However, a limitation lies in the potential interference of other components, like blood in plasma samples. Yet, advancements have been made by combining magnetic particles, significantly enhancing sensor sensitivity and improving the detection of clinical samples, particularly by focusing on TEXs surface markers such as CD63 or CD9 [40]. Exosome-based nano-plasmonic sensors enable label-free profiling, surpassing ELISA and Western blotting in cancer biomarker detection. Droplet digital PCR outperforms traditional methods, detecting low-concentration biomarkers in liquid biopsies [41,42]. This PCR technique, combined with antibody-DNA conjugates and microfluidic devices, efficiently profiles exosomes [43]. It's adept at analyzing exosomes cargo for cancer mutations. Optical assays and electrochemistry-based nanosensors offer rapid, sensitive cancer liquid biopsy analysis. CRISPR-based barcoding deciphers exosome-mediated gene regulation. Multi-omics profiling enhances exosomes analysis, although challenges persist, like isolation protocols, heterogeneity, and tissue-specific diversity. Advanced single-exosome profiling reveals these complexities [44].

## 5 Challenges and future prospective

**Fig. 2:**
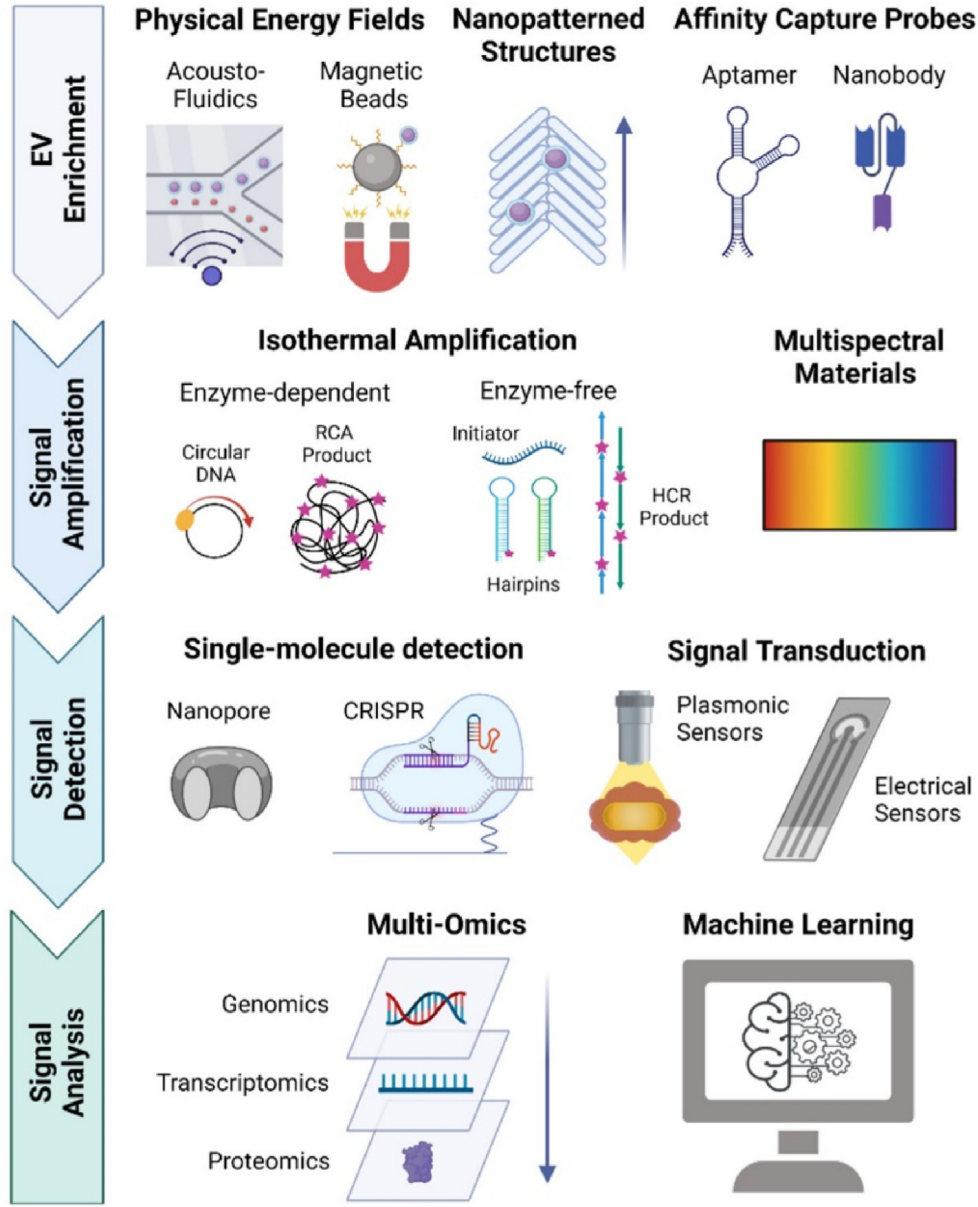
Single Exosomes profiling (Reproduced with permission from ref. [46] Copyright @ 2022 American Chemical Society).

As precision medicine advances, conventional biopsies face limitations, prompting the rise of liquid biopsy as a promising, noninvasive diagnostic tool. Exosomes, vital in physiological and pathological processes, offer potential in clinical applications due to their ubiquitous presence, stability, and specificity. Various biofluids cater to distinct diseases based on anatomy and pathology, like urine for urinary system diseases and blood for tumors and cardiovascular disorders [50]. Yet, bridging basic research with clinical practice poses challenges. The lack of standardizing exosome isolation protocol, exosome heterogeneity, purity, detailed toxicological investigation of therapeutic exosomes, and large-scale production for clinical trials [45,46]. Current technological development and scientific investigation address this issue in more effective ways such as single Exosomes profiling (Fig. 2) [46], exosome barcoding [33,34], and multi-omics profiling of exosomes [47] support to overcome this limitation. Exosome-based cancer liquid biopsy has become a revolutionary era of cancer theranostics.

## 6 Conclusion

The success of treating complex cancers hinges on comprehending the intricate interactions within tumors. Exosomes, small yet influential particles, are increasingly recognized as key mediators cell to cell communication. Exosomes exhibit a diverse cargo, including virions, miRNAs, and proteins, pivotal in various therapeutic contexts. There's a growing interest in leveraging exosomes' potential for delivering therapeutic payloads across biological barriers without invoking immune responses. Nanotechnology has significantly supported exosome research such as detection, characterization, exosome sensor development, and drug loading for successful immunotherapy in clinical trials. However, understanding their impact on distant cell interactions within the heterogeneous tumor microenvironment—crucial in driving drug resistance—remains limited. This article delves into exosome biology in solid tumor resistance, emphasizing their multifaceted role in promoting pathways linked to resistance. Particularly, the role of miRNAs within exosomes is a focal point in ongoing research, presenting a promising avenue for targeting not only tumor cells but the entire tumor microenvironment. As researchers explore deeper, exploring newer strategies harnessing exosomes' context-dependent nature, it's anticipated they will overcome therapeutic resistance, a formidable challenge in cancer therapy. This emerging field holds promise for innovative exosome-based theranostic approaches, offering potential solutions to combat resistant cancers.

## Availability of data and materials

Data sharing does not apply to this article as no datasets were generated or analyzed during the current study.

## Funding

There is no funding for this study.

## Declaration of competing interest

The authors declare that they have no known competing financial interests or personal relationships that could have appeared to influence the work reported in this paper.

## References

[1] Quandt D., Dieter Zucht H., Amann A. (2017). Implementing liquid biopsies into clinical decision making for cancer immunotherapy. Oncotarget.

[2] Aili Y., Maimaitiming N., Mahemuti Y. (2021). The role of exosomal miRNAs in glioma: biological function and clinical application. Front Oncol.

[3] Skotland T., Sandvig K., Llorente A. (2017). Lipids in exosomes: current knowledge and the way forward. Prog Lipid Res.

[4] Puhka M., Takatalo M., Nordberg M.E. (2017). Metabolomic profiling of extracellular vesicles and alternative normalization methods reveal enriched metabolites and strategies to study prostate cancer-related changes. Theranostics.

[5] Kumar S., Dhar R., Kumar L.B.S.S. (2023). Theranostic signature of tumor-derived exosomes in cancer. Med Oncol.

[6] Ghosh S., Dhar R., Gurudas Shivji G. (2023). Clinical impact of exosomes in colorectal cancer metastasis. ACS Appl Bio Mater.

[7] Tai Y.L., Chen K.C., Hsieh J.T. (2018). Exosomes in cancer development and clinical applications. Cancer Sci.

[8] van Niel G., D'Angelo G., Raposo G. (2018). Shedding light on the cell biology of extracellular vesicles. Nat Rev Mol Cell Biol.

[9] Bai S., Xiong X., Tang B. (2020). Exosomal circ_DLGAP4 promotes diabetic kidney disease progression by sponging miR-143 and targeting ERBB3/NF-κB/MMP-2 axis. Cell Death Dis.

[10] Fodor A., Abate B.A., Deák P. (2020). Multidrug resistance (MDR) and collateral sensitivity in bacteria, with special attention to genetic and evolutionary aspects and to the perspectives of antimicrobial peptides-A review. Pathogens.

[11] He L., Chen Y., Ke Z. (2020). Exosomes derived from miRNA-210 overexpressing bone marrow mesenchymal stem cells protect lipopolysaccharide induced chondrocytes injury via the NF-κB pathway. Gene.

[12] Zheng X., Liu J., Li X. (2021). Angiogenesis is promoted by exosomal DPP4 derived from 5-fluorouracil-resistant colon cancer cells. Cancer Lett.

[13] Wang X., Zhang H., Bai M. (2018). Exosomes serve as nanoparticles to deliver anti-miR-214 to reverse chemoresistance to cisplatin in gastric cancer. Mol Ther.

[14] Zhang J., Zhang H.D., Yao Y.F. (2015). β-Elemene reverses chemoresistance of breast cancer cells by reducing resistance transmission via exosomes. Cell Physiol Biochem.

[15] Cao Y.L., Zhuang T., Xing B.H. (2017). Exosomal DNMT1 mediates cisplatin resistance in ovarian cancer. Cell Biochem Funct.

[16] Liu Y., Song B., Wei Y. (2018). Exosomes from mesenchymal stromal cells enhance imatinib-induced apoptosis in human leukemia cells via activation of caspase signaling pathway. Cytotherapy.

[17] Li Y., Liang Y., Sang Y. (2018). MiR-770 suppresses the chemo-resistance and metastasis of triple negative breast cancer via direct targeting of STMN1. Cell Death Dis.

[18] Liu X., Jiang T., Li X. (2020). Exosomes transmit T790M mutation-induced resistance in EGFR-mutant NSCLC by activating PI3K/AKT signalling pathway. J Cell Mol Med.

[19] Wang B., Zhang Y., Ye M. (2019). Cisplatin-resistant MDA-MB-231 cell-derived exosomes increase the resistance of recipient cells in an exosomal miR-423-5p-dependent manner. Curr Drug Metabol.

[20] Ademola H.A., Holmes H., Temilola D.O. (2022). Diagnostic potential of salivary exosomes in oral cancer. Oral Cancer Curr Concepts and Future Perspect.

[21] Wortzel I., Dror S., Kenific C.M. (2019). Exosome-mediated metastasis: communication from a distance. Dev Cell.

[22] Samanta S., Rajasingh S., Drosos N. (2018). Exosomes: new molecular targets of diseases. Acta Pharmacol Sin.

[23] Dakubo G.D., Dakubo G.D. (2016). Cancer biomarkers in body fluids.

[24] Ha D., Yang N., Nadithe V. (2016). Exosomes as therapeutic drug carriers and delivery vehicles across biological membranes: current perspectives and future challenges. Acta Pharm Sin B.

[25] Bu H., He D., He X. (2019). Exosomes: isolation, analysis, and applications in cancer detection and therapy. Chembiochem.

[26] Cappello F., Logozzi M., Campanella C. (2017). Exosome levels in human body fluids: a tumor marker by themselves?. Eur J Pharmaceut Sci.

[27] Schmittgen T.D. (2019). Exosomal miRNA cargo as mediator of immune escape mechanisms in neuroblastoma. Cancer Res.

[28] Li J., Chen J., Wang S. (2019). Blockage of transferred exosome-shuttled miR-494 inhibits melanoma growth and metastasis. J Cell Physiol.

[29] Usman W.M., Pham T.C., Kwok Y.Y. (2018). Efficient RNA drug delivery using red blood cell extracellular vesicles. Nat Commun.

[30] Li M.Y., Liu L.Z., Dong M. (2021). Progress on pivotal role and application of exosome in lung cancer carcinogenesis, diagnosis, therapy and prognosis. Mol Cancer.

[31] Ohno S., Takanashi M., Sudo K. (2013). Systemically injected exosomes targeted to EGFR deliver antitumor microRNA to breast cancer cells. Mol Ther.

[32] Duan H., Liu Y., Gao Z. (2021). Recent advances in drug delivery systems for targeting cancer stem cells. Acta Pharm Sin B.

[33] Wu D., Yan J., Shen X. (2019). Profiling surface proteins on individual exosomes using a proximity barcoding assay. Nat Commun.

[34] Dhar R., Devi A. (2023). Exosomes Barcoding: a smart approach for cancer liquid biopsy. J Liquid Biopsy.

[35] Zhang W., Jiang L., Diefenbach R.J. (2020). Enabling sensitive phenotypic profiling of cancer-derived small extracellular vesicles using surface-enhanced Raman spectroscopy nanotags. ACS Sens.

[36] Zhao Z., Yang Y., Zeng Y. (2016). A microfluidic ExoSearch chip for multiplexed exosome detection towards blood-based ovarian cancer diagnosis. Lab Chip.

[37] Lee K., Fraser K., Ghaddar B. (2018 Jan 23). Multiplexed profiling of single extracellular vesicles. ACS Nano.

[38] Wei H., Ni S., Cao C. (2018 Aug 24). Graphene oxide signal reporter based multifunctional immunosensing platform for amperometric profiling of multiple cytokines in serum. ACS Sens.

[39] Zhuang L., You Q., Su X. (2023). High-performance detection of exosomes based on synergistic amplification of amino-functionalized Fe_3_O_4_ nanoparticles and two-dimensional MXene nanosheets. Sensors.

[40] Boriachek K., Islam M.N., Gopalan V. (2017). Quantum dot-based sensitive detection of disease specific exosome in serum. Analyst.

[41] Olmedillas-López S., Olivera-Salazar R., García-Arranz M. (2022). Current and emerging applications of droplet digital PCR in oncology: an updated review. Mol Diagn Ther.

[42] Bhattacharya B., Dhar R., Mukherjee S. (2023). Exosome DNA: an untold story of cancer. Clin Transl Disc.

[43] Ko J., Wang Y., Carlson J.C.T. (2020). Single extracellular vesicle protein analysis using immuno-droplet digital polymerase chain reaction amplification. Adv Biosyst.

[44] Bordanaba-Florit G., Royo F., Kruglik S.G. (2021). Using single-vesicle technologies to unravel the heterogeneity of extracellular vesicles. Nat Protoc.

[45] Dhar R., Devi A., Patil S. (2023). Exosomes in cancer therapy: advances and current challenges. Electron J Gen Med.

[46] Morales R.T., Ko J. (2022). Future of digital assays to resolve clinical heterogeneity of single extracellular vesicles. ACS Nano.

[47] Kulkarni M., Kar R., Ghosh S. (2024). Clinical impact of multi-omics profiling of extracellular vesicles in cancer liquid biopsy. J Liq Biopsy.

[48] Li S., Yi M., Dong B., Jiao Y., Luo S., Wu K. (2020). The roles of exosomes in cancer drug resistance and its therapeutic application. Clin Transl Med.

[49] Shao H., Im H., Castro C.M. (2018). New technologies for analysis of extracellular vesicles. Chem Rev.

[50] Sonar S. (2024). Extracellular vesicles and cardiovascular disease interlink: a new clinical perspective in cardiology. ClinTransl Disc.

